# Real-Time TEM Observation of the Microstructural Evolution in Silver Nanowires under Heating and Electrical Biasing

**DOI:** 10.1021/acsaelm.5c02254

**Published:** 2026-01-28

**Authors:** Katarzyna Bejtka, Marco Allione, Carlo Ricciardi, Candido Fabrizio Pirri, Gianluca Milano

**Affiliations:** † Department of Applied Science and Technology, 19032 Politecnico di Torino, 10129 Torino, Italy; ‡ Center for Sustainable Future Technologies, Istituto Italiano di Tecnologia, 10144 Torino, Italy; § Advanced Materials Metrology and Life Sciences, INRiM Istituto Nazionale di Ricerca Metrologica, 10135 Torino, Italy

**Keywords:** in situ biasing and heating transmission electron microscopy, Ag NWs, memristive behavior, joule heating and electromigration, rewiring

## Abstract

Silver nanowires (Ag NWs) are of interest for a variety of emerging technologies, such as transparent electrodes, nanoscale heaters, and neuromorphic devices, thanks to their excellent electrical conductivity, flexibility, and tunable nanoscale properties. However, the current understanding of the phenomena underpinning their behavior under electrical stimulation and heating, including failure and reconfiguration effects, is largely based on lab-scale device measurements, offering only indirect insights into the underlying mechanisms. In this work, in situ biasing and heating transmission electron microscopy imaging are performed on individual Ag NWs to directly investigate their morphological and structural evolution under controlled electrical and thermal stress in a vacuum. The results indicate that electrical NW breakdown is dominated by electromigration and localized Joule heating, leading to nanogap formation primarily at the cathode, while thermal decomposition proceeds more gradually along the crystallographic planes. They also provide direct evidence of rewiring phenomena, i.e., the electrically induced reconnection of a previously broken NW, highlighting the self-healing, adaptive, and memristive behavior of the NW under the action of an applied electrical stimulation. Altogether, this work offers fundamental insights into failure and reconfiguration mechanisms at the single NW level, informing the design of Ag NW-based components for flexible electronics, sensors, and neuromorphic systems.

## Graphical abstract

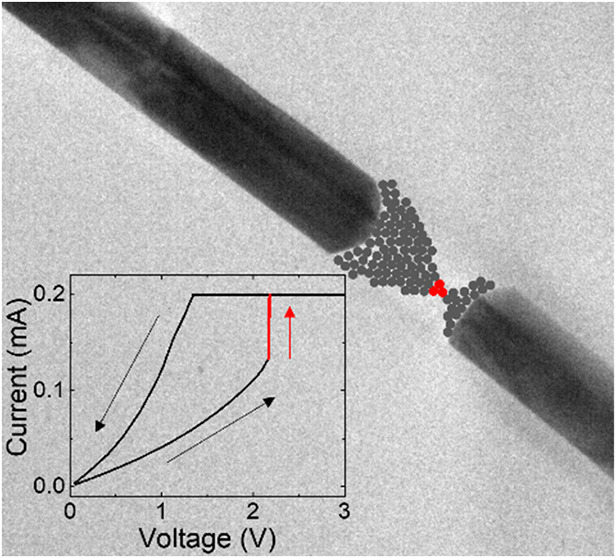

## Introduction

Silver nanowires (Ag NWs) have been exploited for a wide range of applications from transparent electrodes, heaters, and neuromorphic computing applications.^1–6^ They have attracted significant attention for their potential use in such systems due to their excellent electrical conductivity, nanoscale dimensions, and compatibility with low-cost fabrication techniques. In all of these applications, both electrical and thermal properties are crucial for performance and reliability.

In transparent electrodes and heater applications, Ag NW networks often operate at high current densities or elevated temperatures, and it has been shown that NW breakdown events is a source of failure since this can disconnect the percolative pathway, leading to device failure.^7,8^ In neuromorphic computing, complex networks of NWs have been exploited to implement some functionalities analogous to biological synapse by coupling electronics with ionic transport properties and for the implementation of unconventional computing paradigms.^3,9–15^

In this context, morphological evolution of NWs under the application of electrical and thermal stimulation is a crucial aspect affecting device operation across these diverse applications.

Of particular interest are breakdown and rewiring phenomena, during which the conductive path across the single NW can be established and interrupted. The breakdown refers to the rupture of NW under electrical or thermal stress, typically resulting in a nanogap, while rewiring refers to the electrically driven formation of a metallic conductive filament bridging the previously formed gap. Such reconfiguration process offers a mechanism for self-healing and adaptive behavior that have been exploited to emulate synaptic plasticity features in neuromorphic systems,^11^ and affect the reliability of NW-based transparent electrodes and heaters.^16–25^ In particular, it has been shown that breakdown of NWs and subsequent rewiring phenomena are at the base of structural plasticity observed in NW networks, i.e., the change of the network topology depending on the history of applied voltage and current.^26,27^ In this context, breakdown events divide single NWs into two different NW sections connected by a newly generated memristive element, where functionalities rely on rewiring effects. In this scenario, understanding the physical processes behind breakdown and rewiring, including electromigration and filament formation, can help improve the reliability and performance of Ag NW-based devices.

Electrical testing provides only indirect evidence of such phenomena and lacks information on where and how the changes happen, making correlative in situ imaging essential for increased mechanistic understanding. In situ characterization combined with device modeling is believed to be the most efficient approach to achieve a complete and in-depth understanding of the underlying mechanism.^28^ However, the physical origins and conditions that enable reconfiguration are not yet fully understood, and no direct in situ experimental studies of this effect in individual metallic NWs have been reported so far.

While many insightful studies to date have been conducted on larger-scale networks or ensembles^29–31^ and proved in situ approaches to be particularly valuable in understanding the underlying physical mechanisms in nanostructured devices, these configurations primarily probe collective behavior over extended areas. In larger devices, it becomes exceedingly difficult to pinpoint the location of the breakdown or switching. As a result, they do not permit observation of processes taking place within single localized structure, as, for example, NW or at the NW junction. Spatially resolved in situ techniques such as transmission electron microscopy (TEM) can effectively follow individual events in NW or at the NW junction when an ad hoc device fabrication is performed. Studying breakdown and rewiring phenomena in single NWs requires the use of specifically prepared devices where single NWs are contacted with high precision.

Under electrical bias, two primary mechanisms can drive morphological and electrical changes, which lead to the breakdown of metal NWs: Joule heating and electromigration. These mechanisms have been widely investigated in metals such as Cu, Al, and Au, and similar phenomena have been observed in Ag NWs.^32,33^ Real-time in situ TEM imaging, coupled with electrical measurements, has provided valuable insights into how such breakdowns initiate and propagate and has shown that it typically results in voids, necking, or complete fractures at high current densities. Recent studies have provided important insights into the failure mechanisms of Ag NWs under electrical and thermal stress.^6^ Thermal stress can lead to grain boundary diffusion, surface roughening, and Rayleigh-type instabilities,^1^ while electrical stress can accelerate electromigration and localized void formation.^7^ Thermal aging, hot spot formation, and substrate-dependent heat dissipation govern the degradation pathways in Ag NW networks, highlighting the central role of nanoscale mass transport. These studies underline that Ag NW breakdown arises from the interplay between electrical and thermal effects, which are processes that are directly relevant to the microstructural evolution investigated here by real-time TEM.

In addition to purely thermal or electron wind forces, ion migration under an electric field can induce substantial structural transformations, as demonstrated in monocrystalline Cu_2_S NWs by Zhang et al., who observed reversible lattice deformation and spring-like pseudoelastic behavior driven by Cu^+^ ion migration.^34^ This highlights how ionic motion under a bias can strongly influence both electrical and mechanical properties at the nanoscale.

While the breakdown process in Ag NWs has been relatively well studied and in situ observed, the behavior of NW postbreakdown, especially the possibility of re-establishing conductive pathways through rewiring, remains not observed so far. This phenomenon is known to involve electrochemical or thermally assisted processes.^3,35^ Although not previously observed directly in experiments, several works have provided evidence supported by electrical characterization for the feasibility of such phenomena in self-assembled Ag NW networks and suggested that rewiring is related to electrochemical reactions and field-driven ion migration.^3^

This work focuses on in situ observations of Ag NW behavior under electrical and thermal stress, particularly analyzing how breakdown and rewiring occur at the nanoscale. To better understand the interplay between thermally and electrically driven mechanisms, we also studied the evolution of Ag NWs under controlled heating, aiming to correlate structural transitions with temperature. By correlating electrical characteristics with real-time structural evolution via transmission electron microscopy (TEM), we aim to better understand the mechanisms that govern nanogap formation, mass migration, and filament reconnection in Ag NWs. These insights are key to developing more stable and efficient components for nanoscale devices.

## Materials and Methods

### Heating and Biasing In Situ TEM Measurements

The in situ biasing TEM measurements were performed using a Tecnai F20 microscope (FEI), operated at 200 kV acceleration voltage, equipped with a Schottky electron source, an S Twin objective lens, and a Gatan-Orius charge-coupled device (CCD) camera. A high-angle annular dark-field (HAADF) detector was used in scanning TEM (STEM) mode. In situ biasing experiments were conducted using a commercial MEMS-based Fusion holder (Protochips). Electrical measurements were performed in a two-probe configuration with a Keithley 2614b source-measurement unit, applying a voltage sweep with rates ranging from 3.3 to 100 mV s^–1^. In situ breakdown experiments were carried out under vacuum conditions.

The in situ heating TEM was performed using a TALOS F200X microscope (ThermoScientific, Eindhoven, The Netherlands), operated at 200 kV. Images were recorded on a 16 MP CMOS camera. The in situ heating experiments were performed using the commercial MEMS-based NanoEx-i/v holder (ThermoScientific, Eindhoven, The Netherlands), with electrical measurements facilitated by a Keithley 2604B SourceMeter. All in situ experiments were performed under high-vacuum conditions on the order of 10^–7^ mbar.

To minimize electron beam effects to the observed phenomenon, imaging was performed with a low electron dose or by using intermittent illumination. The electron dose was as follows: (i) in situ breakdown experiments were conducted in TEM mode with an illumination dose of ∼50 e^–^ nm^–2^ s^–1^; (ii) rewiring experiments were conducted in STEM mode with a dose per frame of ∼140 e^–^ nm^–2^; and (iii) heating experiments were conducted in STEM mode with a maximum dose per frame of ∼100 e^–^ nm^–2^. The acquisition times and frame rates for all supporting movies 1, 2, 3, and 4 have been added to the corresponding figure captions. Beam-induced effects were evaluated through control experiments. Two types of assessments were performed. First, the influence of the beam on the electrical characteristics was evaluated by monitoring the pristine state of NWs with and without the use of the beam. No changes in the electrical characteristics were observed. In addition, no morphological changes, mass transport, or contrast variations were detected, confirming that the electron beam does not induce any changes in either the electrical characteristics or the material. To avoid any beam-induced thermal effects, the beam was blanked whenever imaging was not performed, and in some experiments, a control NW was observed continuously while the NW of interest underwent intermittent illumination only for final observation.

### Device Preparation

Single-crystalline Ag NWs with a length of 20–50 μm and a diameter of 115 nm (Sigma-Aldrich) were prepared in isopropyl alcohol suspension and were dispersed on commercial e-chips consisting of an electron transparent Si_3_N_4_ (SiN) membrane. Structural characterization of a representative NW is reported in Supporting Figure S1. For biasing experiments, the membrane contained four Pt electrodes with 5 or 10 μm spacing (e-chip E-FED01-LN and E-FEK01-LN, Protochips). Electron-induced Pt deposition was used to bound the NWs to the existing electrodes, performed by Beam-Induced Deposition using a Zeiss Auriga dual-beam system at 1.5 kV. For heating experiments, the NWs were dispersed on commercial e-chips containing a heater and incorporating a SiN membrane for observation.

## Results and Discussion

### NW Breakdown by Biasing

Single Ag NWs have been contacted by dispersing Ag NWs onto commercial MEMS biasing chips designed for in situ TEM. These chips feature prepatterned Pt electrodes on an electron transparent SiN membrane. Figure 1 displays images describing the fabrication process of contacting single Ag NWs, showing the electrode layout on the SiN membrane, single Ag NW bridging two prepatterned electrodes, and a complete device where Pt contacts were deposited via electron beam-induced deposition (EBID) to establish electrical connection. Therefore, this shows a representative as-prepared device prior to any characterization and, thus, before any modifications induced by in situ treatments.

**Figure 1:**
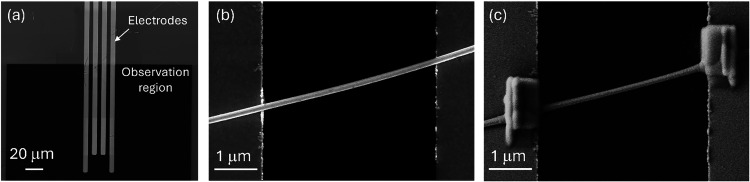
Fabrication of the single Ag NW device, FESEM images of: (a) chip showing the layout of the electrodes on the SiN membrane used as the observation region; (b) a single Ag NW bridging two prepatterned electrodes; (c) a complete device obtained by connecting an Ag NW to a prepatterned electrode by means of EBID deposition of Pt contacts.

Initial electrical characterization confirmed that the Ag NWs exhibit a low pristine state resistance, as shown in Supporting Figure S2, which is consistent with previous studies.^11^ The characterization of pristine states confirms the good electrical contact between the NW and contact pads and serves as a baseline for evaluating the resistance switching behavior induced by electrical stress.

Breakdown events were induced in various single NW-based devices through in situ voltage sweep stimulation and observed using TEM imaging. Device resistance transitions from a low- to high-resistance state can be induced through electrically induced breakdown, and this can be driven by different phenomena, including Joule heating and electromigration, depending on the procedure applied. Distinguishing the roles of these two mechanisms, which could also occur simultaneously but provide different signatures, is essential. Joule heating produces symmetric, thermally driven thinning, while electromigration causes polarity-dependent mass transport, with voids forming at the cathode and hillocks at the anode.^36–39^ In situ TEM experiments give the possibility to observe these effects by examining morphological evolution and by analyzing how the evolution rate varies with the applied voltage sweep. Joule heating is believed to play a critical role in initiating electromigration in Ag NWs, as the localized temperature rise enhances atomic mobility, allowing for mass transport and eventual nanogap formation near the cathode side.^36–39^

As a result, a nanogap is created across the NW or immediately close to the contacts. Despite significant variations in breakdown voltage between devices, all devices exhibited a sudden decrease in the current flow due to electrical breakdown. Figure 2 illustrates a typical breakdown event in two representative devices. This includes the electrical response (*I*–*V* curve), corresponding sequential TEM images showing the morphological evolution of the NW during breakdown highlighting key moments (A, B, and C on the *I*–*V* curve), resulting in the formation of the gap. The final fourth frame shows a higher magnification view of the created gap.

**Figure 2:**
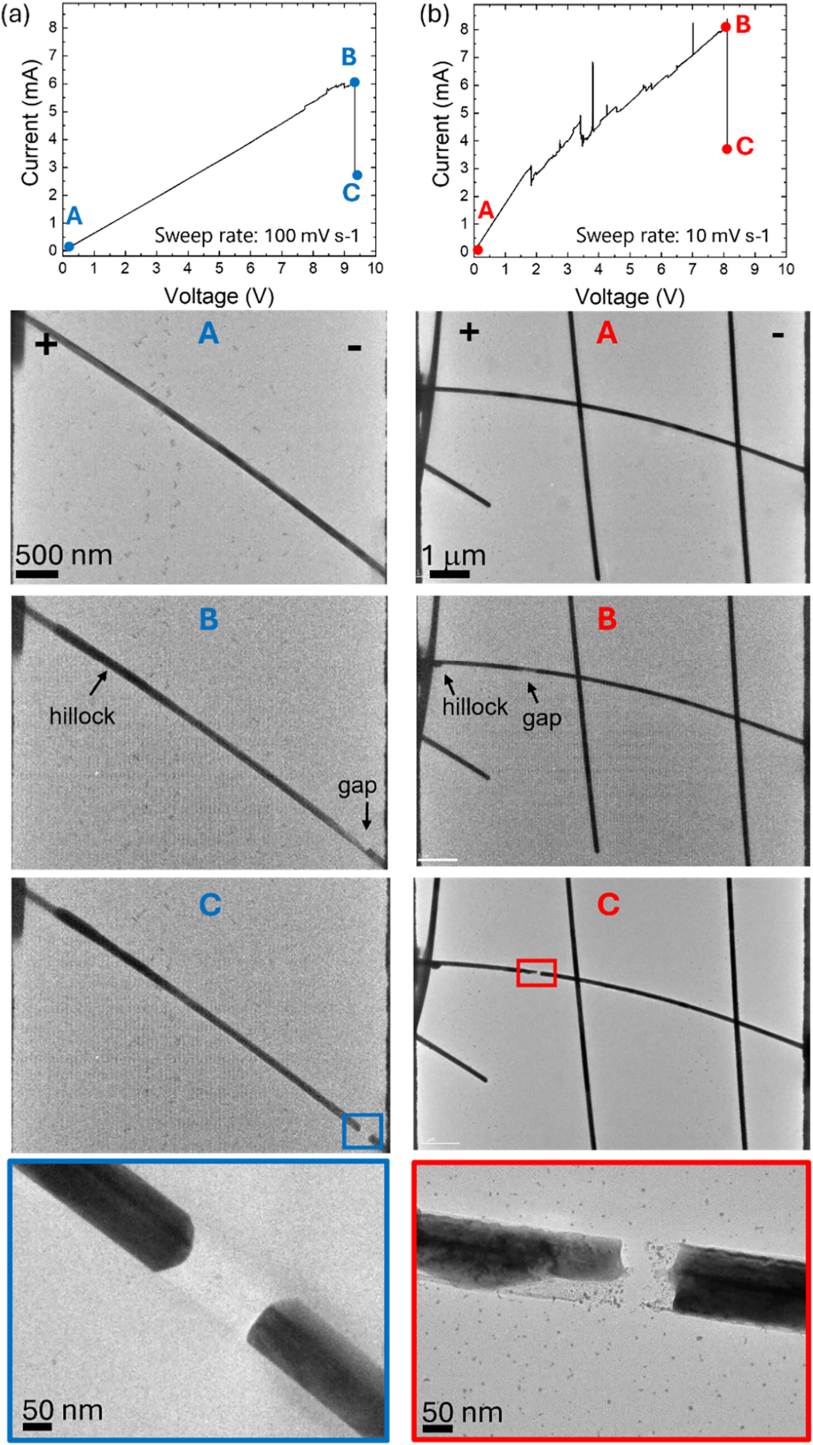
In situ breakdown of Ag NWs during voltage sweep stimulation at voltage sweep rate of (a) 100 mV s^–1^ and (b) 10 mV s^–1^: showing electrical characteristics recorded during the sweep and sequential TEM images showing the corresponding morphological evolution of the Ag NW during electrical breakdown. The arrows indicate the initial creation of the hillocks and gaps.

Bright-field TEM imaging reveals that breakdown initiates with local thinning of the NW at the location where breakdown occurs, followed by the formation of voids, ultimately leading to complete rupture. This occurs predominantly at the cathode side and is consistent with electromigration effects. During breakdown, Joule heating raises the local temperature, which significantly enhances atomic mobility; this raised temperature is essential for electromigration-driven nanogap formation in Ag NWs.^38,39^ The electron wind force, which is related to the momentum transfer between electrons and Ag^+^ ions, dominates over the opposing force of the electric field. This results in silver atoms migrate from the cathode toward the anode. Over time, this results in material depletion at the cathode, leading to void formation and eventual NW breakdown. Electrically, the breakdown manifests as an open circuit due to the loss of material near the cathode.^40,41^ In some cases, however, the breakdown has been reported near the anode, potentially influenced by strongly reduced wind force, which results in the direct force being the dominating electromigration mechanism at the surface of the silver wires.^42^

The morphology of the fracture varies significantly with the applied bias rate, as observed in other works in metallic NWs.^40,43,44^ Depending on the applied bias, the dominant breakdown mechanism can eventually shift from thermal-driven to electro-driven processes. High voltage rate induces rapid thermal runaway, leading to an abrupt and explosive breakdown. This is associated with higher energy input and can lead to partial melting or nanoparticle formation at the breakdown site. It is difficult to study this type of behavior in situ as the explosive breakdown often also damages the membrane, as shown in Supporting Figure S3 in the Supporting Information.

Medium voltage rates (100 mV s^–1^), as applied in this work, result in a more controlled breakdown. Consecutive frames, shown in Figure 2a and the Supporting Movie 1, show the formation of a gap and show that the breakdown started initially slowly but then resulted in suddenly sectioning of the NW. Changes in the contrast along the NW were observed, with time lower contrast is visible at the cathode side, and this is associated with the augmented Ag thinning and atoms migration. Higher contrast is observed at the other end of the NW, finalized with the formation of the hillocks as a result of Ag atoms migration from the cathode toward the anode. After the breakdown, two opposite segments were observed with needle-like clean tips, and there is a sharp gap in between (see the gap in Figure 2a and Supporting Movie 1). The gap shows 5-fold (pentagonal) original NW symmetry at the ends of the needles, suggesting rather rapid material reorganization. It is worth noticing that similar behavior was observed during the in situ heating experiments, where the decomposition of the NWs proceeded along the crystalline planes of preference, as will be shown later within this paper.

At slow bias (10 mV s^–1^), electrically driven processes such as electromigration become more dominant, resulting in the fractures with needle-like morphologies, as shown in Figure 2b and Supporting Movie 2. This is associated with gradual material depletion, and as observed, NW tends to thin gradually before breakdown. The video reveals the dynamic changes occurring under an applied bias. In particular, initial changes in the contrast within the NW result in an irregular structure, and the void formation occurs progressively. Also in this case, the formation of the hillocks was observed at the anode as a result of the migration of Ag atoms from the cathode. Finally, after breakdown, the needles have irregular pointlike shape at both sides.

It is noted that, in some cases, a nonlinear *I*–*V* characteristic can be observed before the breakdown event, as can be observed in Figure 2. This could possibly result from the annealing of contacts and improvement of their quality,^33^ or from slight material redistribution within the NW, where one region thins in the process of electromigration, while another gains mass leading to small variations in resistance. In addition, it can be observed that the current after the breakdown (point C in Figure 2 (a) and (b)) does not reach zero, which is due to the immediate interruption of the voltage sweep when the current drops significantly. In reality, no current is flowing anymore.

It is important to highlight that, in addition to current density, the thermal conductivity of the SiN membrane plays a crucial role in the breakdown process. Due to the intrinsic low thermal conductivity of the SiN membrane, there is not much heat dissipation from the process occurring within the NW. As a result, a significant local temperature rise is expected during electrical stimulation (this aspect will be discussed in more detail in a later section). A slightly different thermal behavior may be expected for the devices fabricated on Si/SiO_2_ substrates, since the SiO_2_ layer has similar thermal conductivity to SiN, but the underlying Si bulk provides thermal sink, enabling more effective heat dissipation than in suspended membranes.

### NW Breakdown by Heating

Breakdown in Ag NWs was also observed during in situ TEM heating experiments, highlighting the impact of the temperature on NW morphology and structure.

As a result of the growth procedure, NW typically had five equivalently flat side surfaces and a characteristic twin boundary along their length.^4,45^ During sample preparation by drop casting, many NWs were found lying with one of the side walls flat against the surface of the e-chip, such that the twin boundary appeared as a straight line in the middle of the NW in TEM images, as seen in Supporting Figure S1. This orientation was frequently observed in our experiments and provided a reference for interpreting the subsequent evolution of the NWs under heating.

Upon heating, morphological changes were observed starting at about 500 °C, which is in agreement with a report of Mayoral et al.^46^ Figure 3 shows that the decomposition, initiated near this temperature, progressively alerts the NW structure.

**Figure 3:**
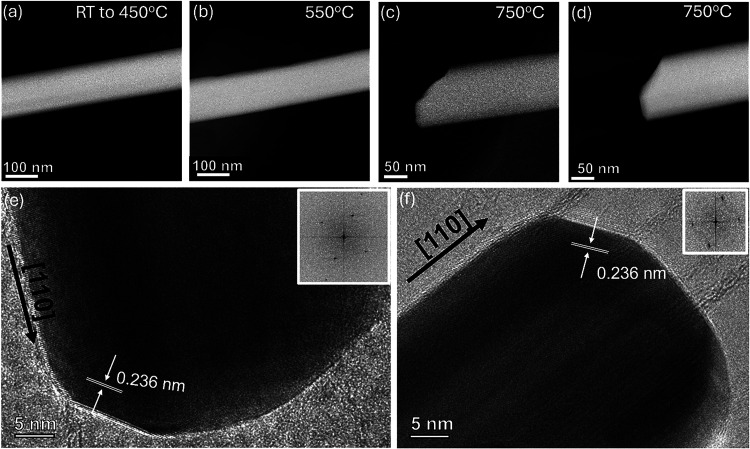
HAADF-STEM images showing the evolution of the morphology of a single Ag NW during heating experiment: (a) at 450 °C, (b) at 550 °C, (c, d) two snapshots of the broken NW taken at two different moments while the heating was paused at 750 °C, and (e, f) two high-resolution TEM images with corresponding FFT analyses, showing that material removal occurs along energetically favorable planes, specifically the {111} plane, which has the lowest surface energy.

At higher temperature, the NW undergoes breakdown or quick decomposition, which proceeds along specific crystallographic orientations, similar to this observed in thinner Ag NWs.^47^ This suggests that the breakdown follows energetically favorable planes. Despite the breakdown, crystallinity was preserved in the remaining NW tip, as seen in both Figure 3c–f and Supporting Movie 3. While Figure 3c,d (and the corresponding Supporting Movie 3) shows that during heating and at the end of heat-induced dissolution, the wire preserves a faceted shape that points toward the idea of a nonhomogeneous dissolution which is more favorable along specific crystallographic directions, this is definitely confirmed in Figure 3e,f. These pictures presents HRTEM images of the NW, confirming the high crystallinity of the tip during decomposition. The process results in the formation of flat and well-defined crystal facets, with an interplanar spacing of 0.236 nm, consistent with the atomic periodicity along the [111] direction. The measured angle between these facets and the nanowire growth direction of approximately 125° confirms this idea, being consistent with the expected crystallographic angle between {111} planes and a [110] growth direction. This process continues also when the heating is paused, and the NW remains at the constant temperature. This contrasts with most of the bias-induced breakdowns studied, where the current flow ceases upon rupture, halting further morphological evolution. In contrast, heating-induced breakdown allows continued change due to the sustained thermal energy input.

### Postbreakdown NW Consumption

In several instances, it was observed that the bias-induced breakdown did not result in a halting of morphological evolution. As can be observed in Figure 4, after the NW rupture and nanogap formation, and therefore when the current flow ceases, the NW undergoes progressively material depletion from the gap area, and its accumulation on the anode side. In the following step, there is consumption of the NW from both sides. The fact that it initiates on one side suggests the presence of a hot spot, and therefore this phenomenon suggests structural transformation driven by residual thermal energy. Immediately before the breakdown, the local hot spot related to Joule heating is expected to be strongest at the position within the NW where electromigration concentrates the current. After breakdown, this region cools gradually due to the limited heat sinking through the membrane. This results in heat redistribution and creation of the thermal gradient along the NW in an asymmetric way. The heat is first localized at the NW close to the gap and therefore close to cathode and expand toward anode. This gradient can result in continued surface diffusion or sublimation-like material transport even after the current flow has stopped. Such behavior was observed for quicker biasing and contrasts with breakdown under slower biasing, where the process stops immediately at the moment of the NW breakdown.

**Figure 4:**
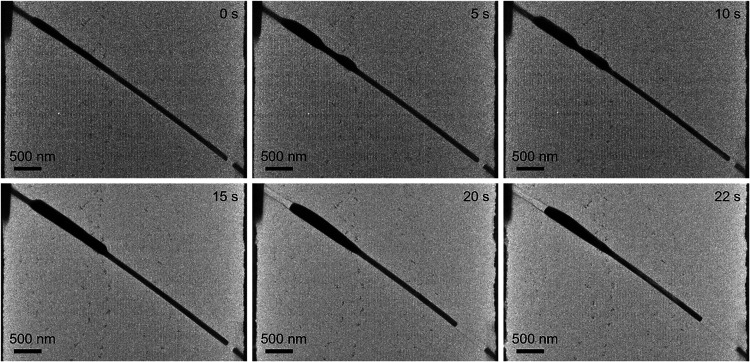
Sequential TEM images showing the morphological evolution of the Ag NW after electrical breakdown under high-biasing-rate conditions. Time shown indicates the elapsed time after the moment of NW rupture (*t* = 0 s).

These results show the importance of bias-rate control and thermal management in device design, which is particularly of high importance in neuromorphic applications, where network reconfiguration plays a central role.^3^

### NW Rewiring

Rewiring effects in Ag NW networks are of particular importance in neuromorphic applications, as they can enable long-term modifications of network topology, emulating structural plasticity effects typical of biological neuronal circuits.^3^ In the context of memristive and neuromorphic device architectures, rewiring phenomena in metallic NWs are of particular interest due to their potential to enable self-healing, adaptive connectivity, and reconfigurable circuit behavior. Understanding and controlling such processes are essential for developing systems that can dynamically respond to electrical stimuli and recover functionality after failure.

In devices subjected to an even slower biasing rate of 3.3 mV s^–1^, the breakdown resulted in a narrower gap, as can be observed in Figure 5a. In this case the NW failed at 2.1 V. The breakdown occurred close to one of the electrodes, specifically to the cathode, with depleted silver accumulating at the anode, which is consistent with electro-breakdown. A tapered Ag tip was observed at the anode side, while only the Pt contact remained at the cathode. This composition was confirmed by EDX analysis, as shown in Figure 5c.

**Figure 5:**
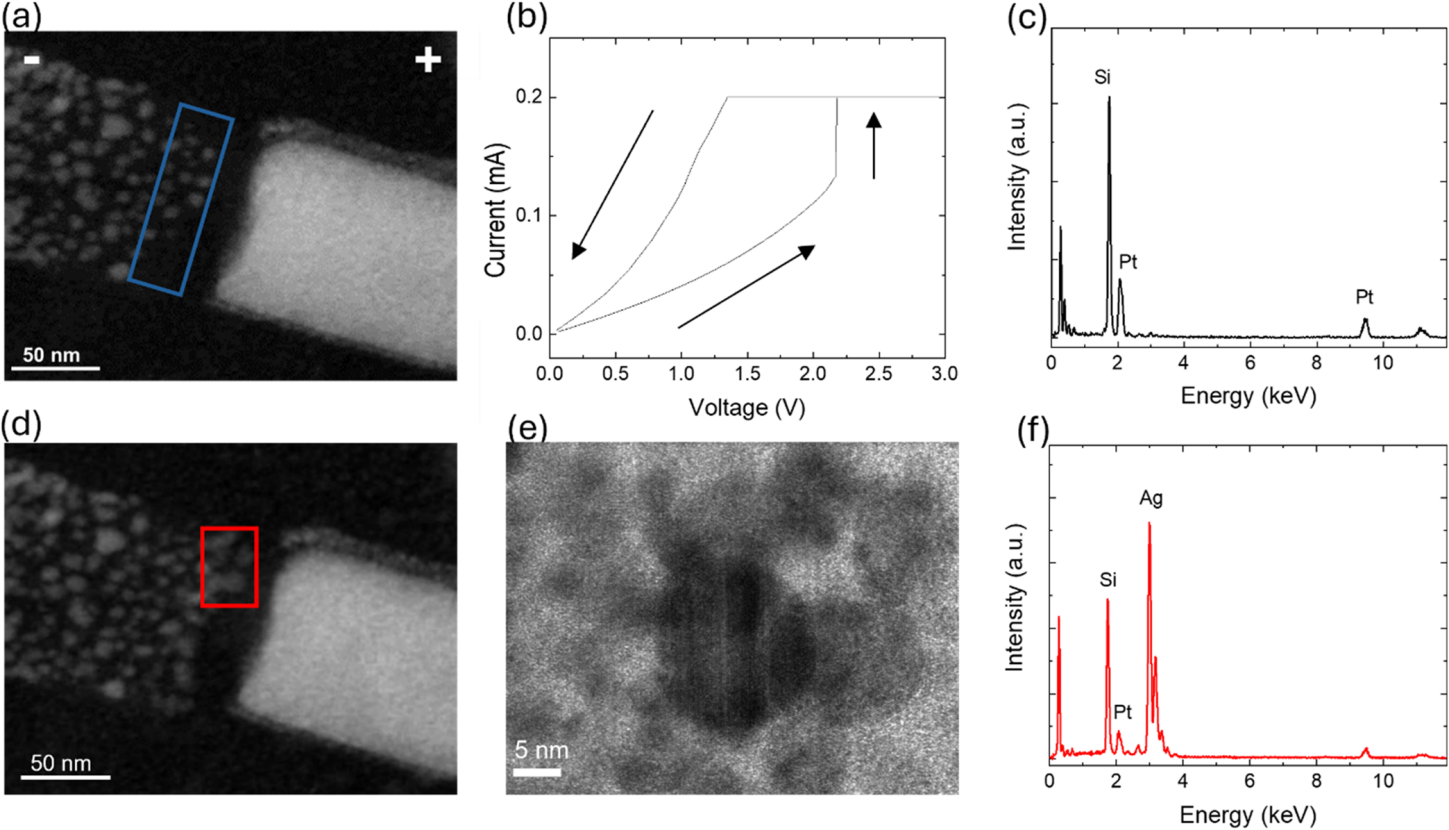
(a) STEM image of a nanogap across the NW after electrical breakdown. (b) Voltage sweep induced NW rewiring. (c) EDX spectrum before and after rewiring acquired from the area indicated in panel (a). (d) STEM images after rewiring. (e) Structural characterization after rewiring. (f) EDX spectrum after rewiring acquired from the area indicated in (b).

After breakdown, the electrical connection can be re-established through an electrochemical rewiring process.^48^ This can be performed under a voltage sweep, as shown in Figure 5b. A sudden increase in current indicates the formation of a conductive filament bridging the nanogap. This results in lowering the gap resistance and turning the device to a lower resistance state. This process can involve electrochemical dissolution of the positively biased Ag NW needle, releasing Ag^+^ ions that migrate across the nanogap under the applied electric field and subsequently nucleate and form a conductive filament. The non-ohmic decrease in current observed upon voltage reduction suggests that the conductive path is not a continuous metallic wire but rather a discontinuous filament.

In situ STEM observation (Figure 5d and Supporting Movie 4) of this process provides direct evidence supporting this interpretation as it revealed the appearance of new particles on the anode side. This is consistent with filament formation growing from the inert electrode toward Ag NW, coinciding with the moment when a sudden increase of the current was observed. The cations, which diffuse, continue to become reduced at the end, and therefore the growth of the particles is observed. The electric field, which is highest at the front of the newly created structure possibly leads to continued growth at the end of the newly formed structures. This behavior is in agreement with mechanisms observed in electrochemical metallization (ECM) cells, where filament formation is driven by the dissolution and migration of the active electrode material into the dielectric layer and their reduction at the opposite electrode, which is related to the formation of the initial filament. However, as this experiment is run in high vacuum, electrochemical phenomena underlying resistive switching effects are expected to be hampered. This is consistent with previous experimental and theoretical observation that water molecules related to ambient humidity reduce the energy barrier for Ag^+^ ion migration, facilitating the conductive filament formation.^49–51^ It has been also shown that resistive switching can be suppressed in vacuum conditions.^49^ However, it should be pointed out that here reported results obtained in vacuum are qualitatively consistent with results obtained by measuring devices in air.^11^

The crystal structure of the newly grown particles within the formed filament was studied by high-resolution TEM (HRTEM) (shown in Figure 5e) and EDX spectroscopy (shown in Figure 5f). These techniques confirmed that the newly formed particles composing conducting filament are metallic Ag, and are crystalline. The filament is composed of a series of nanoparticles, some of these appear to be separated by gaps of the nanometer scale. It is difficult to determine from the STEM live-acquisition images whether the nanoparticles are fully separated or occasionally in point contact, as this remains below spatial resolution. Even though the filament is composed of nanoparticles and although it is not in the form of a continuous wire, it still allows conductance in the on-state. This can arise from charge transport through nanogap tunneling or through point contacts between nanoparticles, which are mechanisms known to sustain conduction in nanoparticle-based systems,^52,53^ and is also consistent with quantum-conductance effects previously observed in Ag nanowire nanogaps.^11^

## Summary and Conclusions

This study provides insights into the in situ evolution of morphological properties of individual Ag NWs subjected to electrical bias stimulation or thermal ramping in a vacuum, which is of huge interest for the development of NW-based neuromorphic systems as well as for transparent electrodes and heaters.

We investigated the breakdown mechanisms of Ag NWs under electrical and thermal stress, demonstrating that electrical and structural transitions occur through distinct mechanisms. Electrical breakdown is governed by localized Joule heating and electromigration, leading to rapid nanogap formation predominantly at the cathode side, while thermally induced decomposition proceeds gradually along the crystallographic planes at elevated temperatures.

The simultaneous in situ TEM observation and electrical stimulation of the same NW allows for a direct correlation between the electrical response and real-time structural evolution. This analysis revealed that some morphological signatures, such as needle-like fracture tips and consumption along crystalline planes related to 5-fold symmetry, were similar for both stimulation modes, pointing to underlying material reorganization pathways, which are of interest to resistive switching behavior.

The behavior observed during in situ heating of the NWs shows that an NW undergoing breakdown induced solely by temperature continues to change its morphology, even after the breakdown is complete. In contrast, during bias-induced breakdown, the hot spot is undoubtedly linked to the current flow, which stops once the NW undergoes breakdown, preventing further morphological changes.

Such insights are particularly useful for the design of nanoscale devices requiring precise control over electrical, thermal, and structural properties, including applications such as transparent electrodes, sensors, flexible electronics, and neuromorphic NW networks. In the latter, Ag NW-based elements are increasingly explored for their memristive behavior, where understanding the precise dynamics of nanogap formation, atomic migration, and filamentary conduction under different stress conditions is critical for designing reliable, reconfigurable, and energy-efficient Ag NW-based components for next-generation electronics.

## Supplementary Material











## Notes

TEM characterisation of the representative Ag NW; pristine state *I*–*V* characteristics of single Ag NW memristive devices; in situ breakdown of Ag NW during voltage sweep stimulation with its electrical characteristics recorded during the sweep; TEM images showing the initial state and the final state; EDX compositional characterization of the tested structure after the rewiring (PDF)
BF-TEM movie illustrating the breakdown of the Ag NW under application of bias until the breakdown moment at 100 mV s-1 (Movie 1) (MP4)
BF-TEM movie illustrating the breakdown of the Ag NW under application of bias until the breakdown moment at 10 mV s-1 (Movie 2) (MP4)
HAADF-STEM movie showing progressive consumption of the Ag NW during the heating experiment (Movie 3) (MP4)
HAADF-STEM movie illustrating the formation of the conductive bridge after the break down, between the cathode and the anode (Movie 4) (MP4)
