# Supplementary material for: Real-Time TEM Observation of the Microstructural Evolution in Silver Nanowires under Heating and Electrical Biasing
Source: ACS Appl Electron Mater. 2026 Jan 28;8(3):1156–65. doi: 10.1021/acsaelm.5c02254 (PMC12895405; doi:10.1021/acsaelm.5c02254)
Supplement: Supplementary file 1 [file el5c02254_si_001.pdf]

Supporting Information

Real-time TEM observation of the microstructural evolution in silver nanowires under heating and electrical biasing.

Katarzyna Bejtka[1,2,], Marco Allione[1], Carlo Ricciardi[1], Candido Fabrizio Pirri[1,2], and Gianluca Milano[3]*

[1]Department of Applied Science and Technology, Politecnico di Torino, 10129 Torino, ITALY

[2]Center for Sustainable Future Technologies, Istituto Italiano di Tecnologia, 10144 Torino, ITALY

[3]Advanced Materials Metrology and Life Sciences, INRiM Istituto Nazionale di Ricerca Metrologica, 10135 Torino, ITALY

(corresponding author: katarzyna.bejtka@polito.it)*

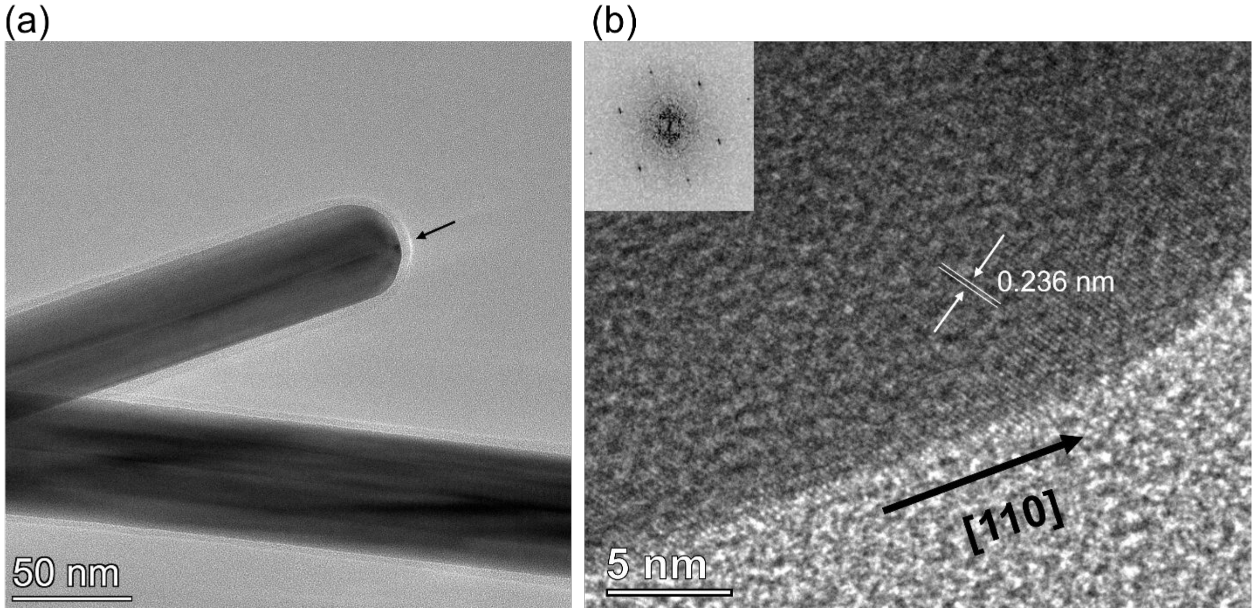

Supplementary Figure S1. TEM characterisation of the representative Ag NW: Bright-field TEM image, with the arrow indicating a twin plane situated parallel to Ag nanowire's longitudinal axis (a) and High Resolution TEM with corresponding FFT transformation indicating the growth direction of the Ag NW is along [110] as shown by the arrow (b).

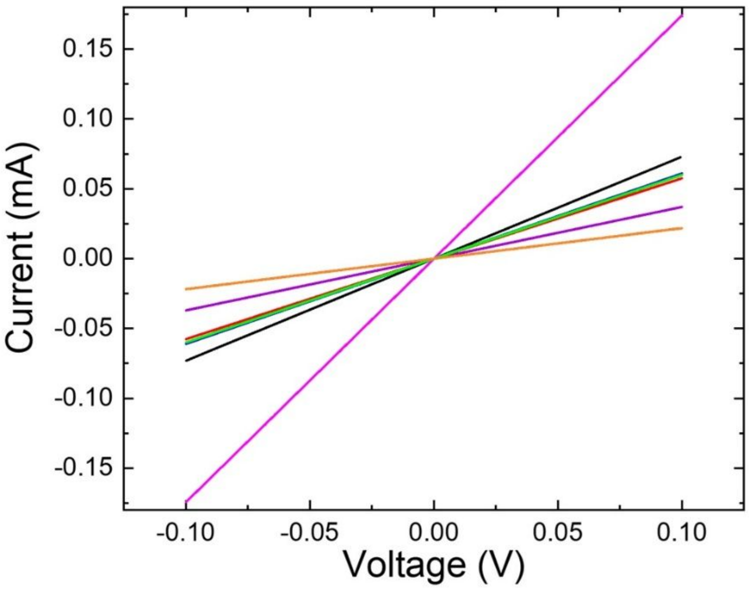

Supplementary Figure S2. Pristine state *I–V* characteristics of single Ag NW memristive devices, with resistance values in the range of 570 – 4500 Ω.

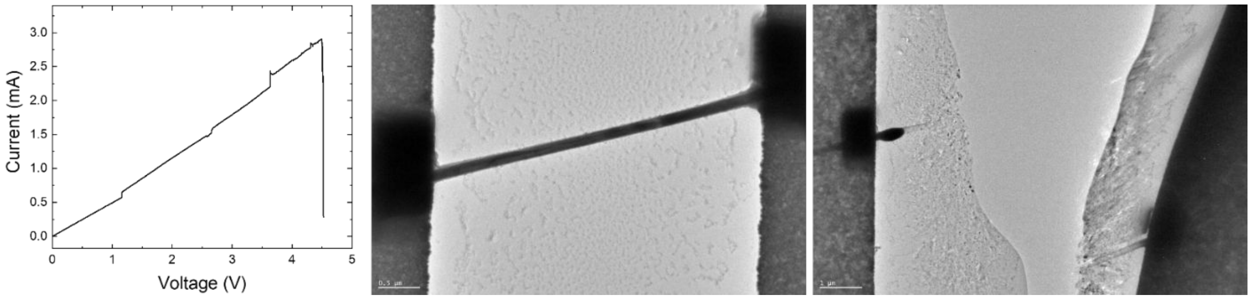

Supplementary Figure S3. *In situ* breakdown of Ag NW during voltage sweep stimulation: (a) Electrical characteristics recorded during the sweep and (b) TEM images showing the initial state and the final state when the NW underwent the breakdown and the membrane broke.

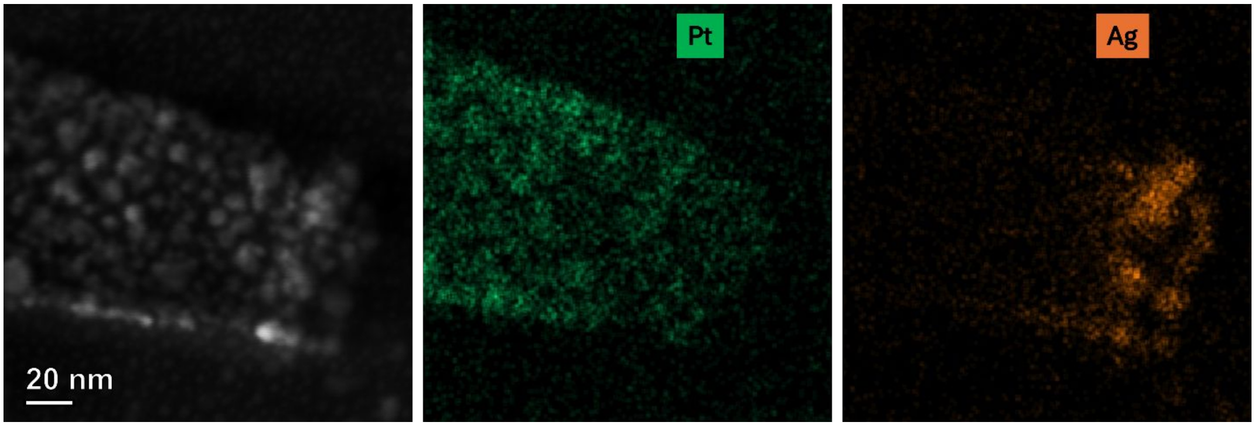

Supplementary Figure S4. Compositional characterization of the tested structure after rewiring and some additional stress provided to the Ag NW: HAADF STEM image, and EDS mapping showing the distribution of Pt (green) and Ag (orange). This characterization shows that the particles created during the rewiring process are Ag.

Supplementary Movies: list and details

Supplementary Movie 1

Description: BF-TEM movie illustrating the breakdown of the Ag nanowire under application of bias until the breakdown moment, in detail in the range 5.4 V to 9.3 V at 100 mV s^{-1}. The illumination was held constant at approximately 50 e^{-} nm^{-2} s^{-1}, with frame rate of 8 images per second.

The movie playback rate is real time.

Supplementary Movie 2

Description: BF-TEM movie illustrating the breakdown of the Ag nanowire under application of bias until the breakdown moment, in detail in the range 7.4 V to 8.1 V at 10 mV s^{-1}. The illumination was held constant at approximately 50 e^{-} nm^{-2} s^{-1}, with frame rate of 8 images per second.

The movie playback rate is 2 times real time.

Supplementary Movie 3

Description: HAADF-STEM movie showing progressive consumption of the Ag NW during the heating experiment, recorded while the heating was paused and the temperature was held at 750 ^{o}C. The dose per frame was at most at the value of ~100 e^{-} nm^{-2}, with frame time of approximately 2.5 seconds.

The movie playback rate is 10 times real time.

Supplementary Movie 4

Description: HAADF-STEM movie illustrating the formation of the conductive bridge after the break down, between the cathode and the anode. The forming was induced by voltage sweep performed at 20 mV s^{-1}.

The dose per frame was kept at ~140 e^{-} A^{-2}, with frame rate of 3 images per second.

The movie playback rate is real time.
